# Evaluation of [18F]PSMA‐1007 uptake variability in patients with prostate cancer

**DOI:** 10.1111/cpf.70016

**Published:** 2025-06-15

**Authors:** Elin Trägårdh, Måns Larsson, David Minarik, Olof Enqvist, Lars Edenbrandt

**Affiliations:** ^1^ Department of Clinical Physiology and Nuclear Medicine Skåne University Hospital Malmö Sweden; ^2^ Department of Translational Medicine and Wallenberg Center for Molecular Medicine Lund University Malmö Sweden; ^3^ Eigenvision AB Malmö Sweden; ^4^ Department of Radiation Physics Skåne University Hospital Malmö Sweden; ^5^ Chalmers University of Technology Gothenburg Sweden; ^6^ Institute of Medicine, Sahlgrenska Academy Gothenburg University Gothenburg Sweden

**Keywords:** [18F]PSMA−1007, PET, tumour sink effect, prostate cancer, PSMA, radioligand therapy

## Abstract

**Background:**

The biodistribution of PSMA‐ligands is of interest in radionuclide therapy planning. We investigated the variability of [^18^F]PSMA‐1007 uptake in organs at risk and in relation to tumour burden in prostate cancer patients.

**Methods:**

A total of 1086 patients who underwent PSMA PET‐CT for staging or recurrence of prostate cancer were included. Total lesion volume (TLV) and total lesion uptake (TLU) were calculated from manual segmentations. The mean standardized uptake value (SUV_mean_) in the organs at risk kidneys, liver, parotid glands and spleen was obtained. Correlations between TLV/TLU and SUV_mean_ in normal tissues were calculated using Spearman rank correlation. SUV_mean_ in normal tissues was stratified into groups based on TLV.

**Results:**

The median (IQR) SUV_mean_ of the kidneys, liver, parotid glands, and spleen was 13.1 (IQR 4.6), 11.8 (4.4), 18.6 (6.8) and 11.3 (5.8), respectively. The median TLV was 3.8 cm^3^ (9.7) and median TLU was 31.2 cm^3^ (106.3). There was no significant correlation between TLV or TLU and SUV_mean_ for the liver, parotid glands, or spleen, but a weak negative correlation between TLV/TLU and SUV_mean_ in the kidneys (*r* = −0.011, *p* = 0.0005; *r* = −0.09, *p* = 0.003). There was a tendency towards a lower SUV_mean_ in the kidneys and parotid glands in patients with a very high TLV.

**Conclusions:**

There was a large uptake variability in organs at risk, which demonstrates the need for individual pretherapy dosimetry. There may be a tumour sink effect in the kidneys and parotid glands in patients with a very high TLV.

## 1 INTRODUCTION

Theragnostics is the concept of combining therapeutics with diagnostics, where a diagnostic test can identify patients most likely to benefit from a specific treatment. Recent developments in nuclear medicine include radiolabelled PSMA‐ligands for prostate cancer (e.g. [^68^Ga]Ga‐PSMA‐11, [^18^F]PSMA‐1007 or [^18^F]DCFPyL used for diagnostics and [^177^Lu]Lu‐PSMA‐617 for therapy) (Ahmadzadehfar et al., 2016; Ferdinandus et al., 2017; Hofman et al., 2018; Kabasakal et al., 2015; Yordanova et al., 2017). Radiopharmaceuticals are often not specific for the cancer in question; for example, PSMA ligands also accumulate in other tissues such as the liver, spleen, kidneys and salivary glands (Giesel et al., 2017; Hvittfeldt et al., 2022). These organs exhibit high variability of tracer uptake (Hvittfeldt et al., 2022). Decreased uptake in healthy tissue (organs at risk) in the presence of a large tumour burden, due to decreased bioavailability in healthy tissue, has been described (Beauregard et al., 2012; Viglianti et al., 2018). This phenomenon has been called “the tumour sink effect” and has been discussed in connection with radioiodine therapy of thyroid carcinoma (Basu et al., 2020), in [^177^Lu]Lu‐DOTATATE RNT of neuroendocrine tumours (Beauregard et al., 2012), and recently also in patients with prostate cancer, treated with [^177^Lu]Lu‐PSMA‐617 (Filss et al., 2018; Gafita et al., 2022). Lower uptake in organs at risk in a pre‐therapy diagnostic image may translate into lower healthy tissue irradiation in patients receiving RNT. Therefore, there might be room for an individualized (higher) dosage of the therapeutic radiopharmaceutical, instead of the commonly used strategy of giving a fixed amount of activity.

The relation between uptake in organs at risk and tumour burden has been investigated for PSMA PET‐CT imaging for the radiopharmaceuticals [^68^Ga]Ga‐PSMA‐11 and [^18^F]DCFPyL, with varying results. Gafita et al. (Gafita et al., 2022) showed that patients with a very high tumour volume in [^68^Ga]Ga‐PSMA‐11 images had a significantly lower uptake in the organs at risk (kidneys, spleen, salivary glands and liver), with a moderate correlation. Gaertner et al. (Gaertner et al., 2017) also found a significant reduction in tracer uptake in dose‐limiting organs in patients with high tumour burden on [^68^Ga]Ga‐PSMA‐11 images. Werner et al. (Werner et al., 2020) found no such correlation on [^18^F]DCFPyL images. To the best of our knowledge, no studies on the variability of radiopharmaceutical uptake in organs at risk or the relation between uptake in organs at risk and tumour burden exist for [^18^F]PSMA‐1007.

The aim of the present study was to retrospectively analyse the variability of uptake of the radiolabelled PSMA‐ligand [^18^F]PSMA‐1007 in organs at risk, and secondary to investigate the relationship between the uptake in organs at risk and tumour burden, in patients who underwent PET‐CT for the management of prostate cancer.

## 2 MATERIALS AND METHODS

### 2.1 Patients

A total of 1086 patients who performed a clinically indicated [^18^F]PSMA‐1007 PET‐CT at Skåne University Hospital, Lund or Malmö, Sweden were included. Indications for performing a PSMA PET‐CT at Skåne University Hospital are primary staging of newly diagnosed high‐risk prostate cancer or secondary staging due to biochemical recurrence. All patients provided written informed consent to participate in the study.

### 2.2 PET‐CT

Patients were injected with a mean of 4.0 (standard deviation (SD) 0.2) MBq/kg of [^18^F]PSMA‐1007 via intravenous injection through the Iris radiopharmaceutical injector (Comecer, Bologna, Italy), with an accuracy of ± 3% of the administered activity, verified using a Fidelis secondary standard radionuclide calibrator (NPL, n.d). Image acquisition started after 2 h. Images were obtained from mid thighs to the base of the skull. A CT scan was acquired for attenuation correction. Images were acquired using GE Healthcare Discovery MI scanners and followed the EANM procedure guidelines for prostate cancer imaging (Fendler et al., 2023). SUV was calculated as

$$\mathrm{SUV}=\frac{\mathrm{Ac}}{\mathrm{Ai}/w}$$

where *Ac* is the activity per unit volume in the image, *Ai* is the administered activity, decay corrected to the time of imaging, and *w* is the patient weight. Assuming a patient density of 1 g/cm^3^, the SUV parameters become unitless.

### 2.3 Calculation of tumour burden

One experienced nuclear medicine physician manually segmented tumour‐related uptake using the RECOMIA platform. Total lesion volume (TLV) was calculated as a sum of all tumour segmentations in a patient. Total lesion uptake (TLU) was calculated as TLV multiplied by the SUV_mean_ in the corresponding volume. The percentage of the activity in the tumour in relation to the total amount of injected activity was calculated as TLU/weight.

### 2.4 Uptake measurement in organs at risk

Following the methodology outlined in (Gafita et al., 2022), uptake was measured in the parotid glands, kidneys, liver, and spleen; organs known to exhibit high uptake levels (Hvittfeldt et al., 2022). The liver and spleen were automatically segmented using a convolutional neural network (CNN) that processed the corresponding CT images as input (Edenbrandt et al., 2022). For the parotid glands and kidneys, task‐specific CNNs were trained, utilizing both the CT and PET images as input. The parotid gland CNN was trained on a manually annotated data set comprising 21 studies, while the kidney CNN, which segmented only the kidney parenchyma, was trained on 175 studies. When the automatic segmentations failed, they were manually corrected by an experienced nuclear medicine physician. For each organ segmented automatically, SUV_mean_ was measured within a spherical volume of interest (VOI) placed inside the segmentation. Sphere diameters were 30 mm for the liver and 15 mm for the other organs. The sphere's location was determined automatically using an algorithm designed to avoid regions with metastases and edge effects. Patients with a high tumour burden were manually checked to ensure no tumour were included in the organs at risk VOIs. The process for determining the measurement location was as follows:

1. Calculate SUV_peak_ for all pixels in normal organ (measured in a spherical 1 cm^3^ VOI).
2. For each pixel in the organ, center the measurement sphere at the pixel and calculate both the maximum and standard deviation of SUV_peak_ values within the sphere. Exclude pixels where any part of the sphere is within 5 mm of the organ's edge.
3. Calculate median of (maximum + standard deviation) SUV_peak_ for all pixel in normal organ.
4. Choose center of measurement volume as pixel with (maximum + standard deviation) SUV_peak_ closest to median.

### 2.5 Statistical analysis

Values were reported as mean ± SD or median and interquartile range (IQR). Correlations between TLV or TLU and uptake in organs at risk were evaluated using the Spearman correlation coefficient with a 2‐tailed test for significance. A p‐value of 0.05 or less was considered statistically significant. For scatter plot visualization, TLV and TLU were logarithmized after adding 1. For easier comparison with the results by Gafita et al. (Gafita et al., 2022), SUV_mean_ in organs at risk were stratified by TLV using the same cut‐offs. The six groups were patients with TLV = 0, >0–25 cm^3^ (very low), >25–189 cm^3^ (low), >189–532 cm^3^ (moderate), >532–1355 cm^3^ (high) and >1355 cm^3^ (very high). Analyses were performed using Microsoft Excel 365.

### 2.6 Artificial intelligence created content

No large language models were used in the development of the manuscript.

## 3 RESULTS

### 3.1 Patient characteristics

Characteristics for the included 1086 patients are shown in Table 1. The spleen was missing in one patient (splenectomy), and the parotid glands were missing in 5 patients (not included in the field of view).

**Table 1:** Patient characteristics.

Patient characteristics	
Age (y)	70 ± 7 (41–89)
Weight (kg)	87 ± 14 (48–146)
Height (cm)	177 ± 7 (156–198)
Injected activity (MBq/kg)	4.0 ± 0.2 (2.8–6.0)
Primary staging (*n* (%))	693 (64%)
Recurrence (*n* (%))	393 (36%)

*Note*: Values are shown as mean ± SD (range), unless otherwise stated.

### 3.2 Organs at risk and tumour measurements

**Figure 1:**
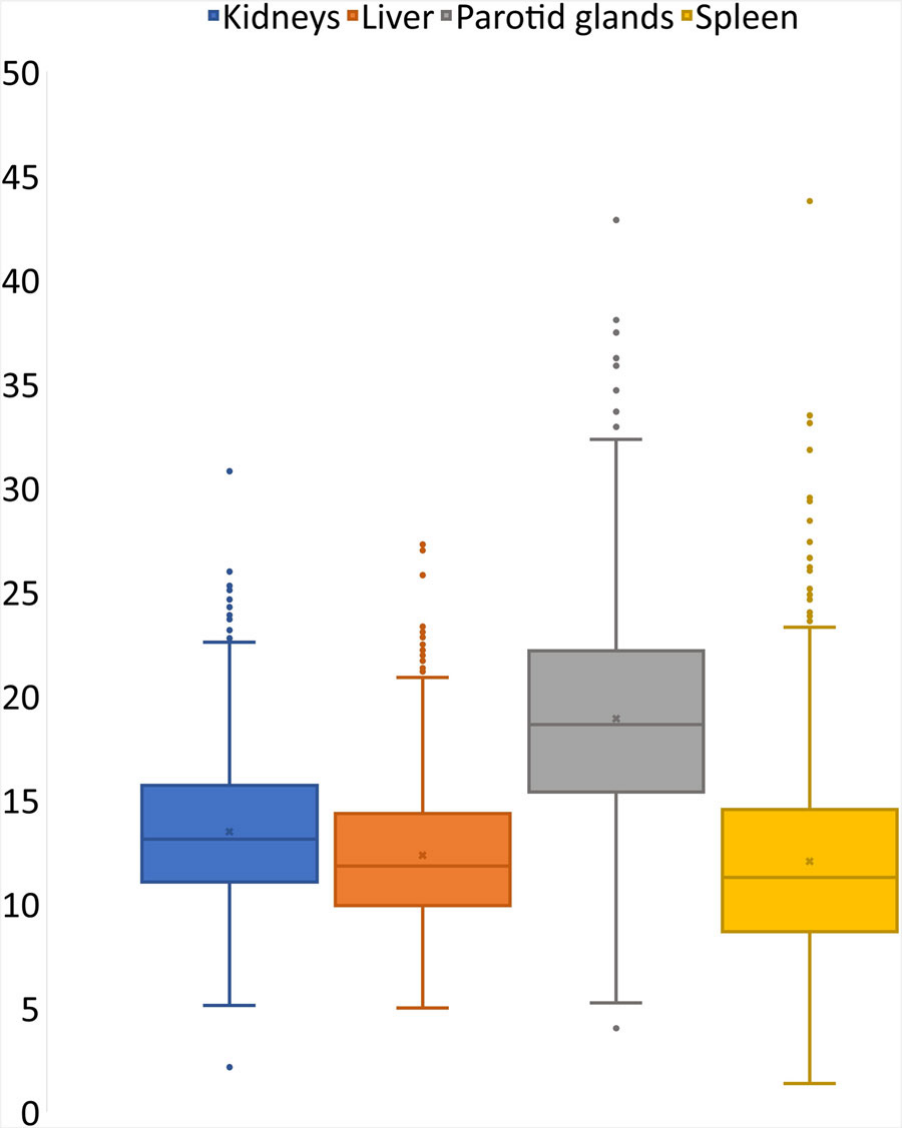
SUVmean of reference organs shown as boxplots. The horizonal line in the box represents the median. The box represents the 1st and 3rd quartiles.

The median (IQR) SUV_mean_ of the kidneys, liver, parotid glands, and spleen was 13.1 (4.6), 11.8 (4.4), 18.6 (6.8) and 11.3 (5.8), respectively (Figure 1). A total of 157 patients had no tumorous lesions. The median TLV for patients with tumour was 3.8 cm^3^ (IQR 9.7 and range 0.06–2066 cm^3^) and median TLU was 31.2 cm^3^ (IQR 106.3 and range 0.23–17 512 cm^3^)). The median percentage of TLU of those with tumour in relation to total injected activity was 0.039% (IQR 0.12%, range 0.001–18.6%). Only three of the patients had more than 10% of the total injected activity located within the tumours.

### 3.3 Correlations between tumour burden and organ at risk uptake

**Figure 2:**
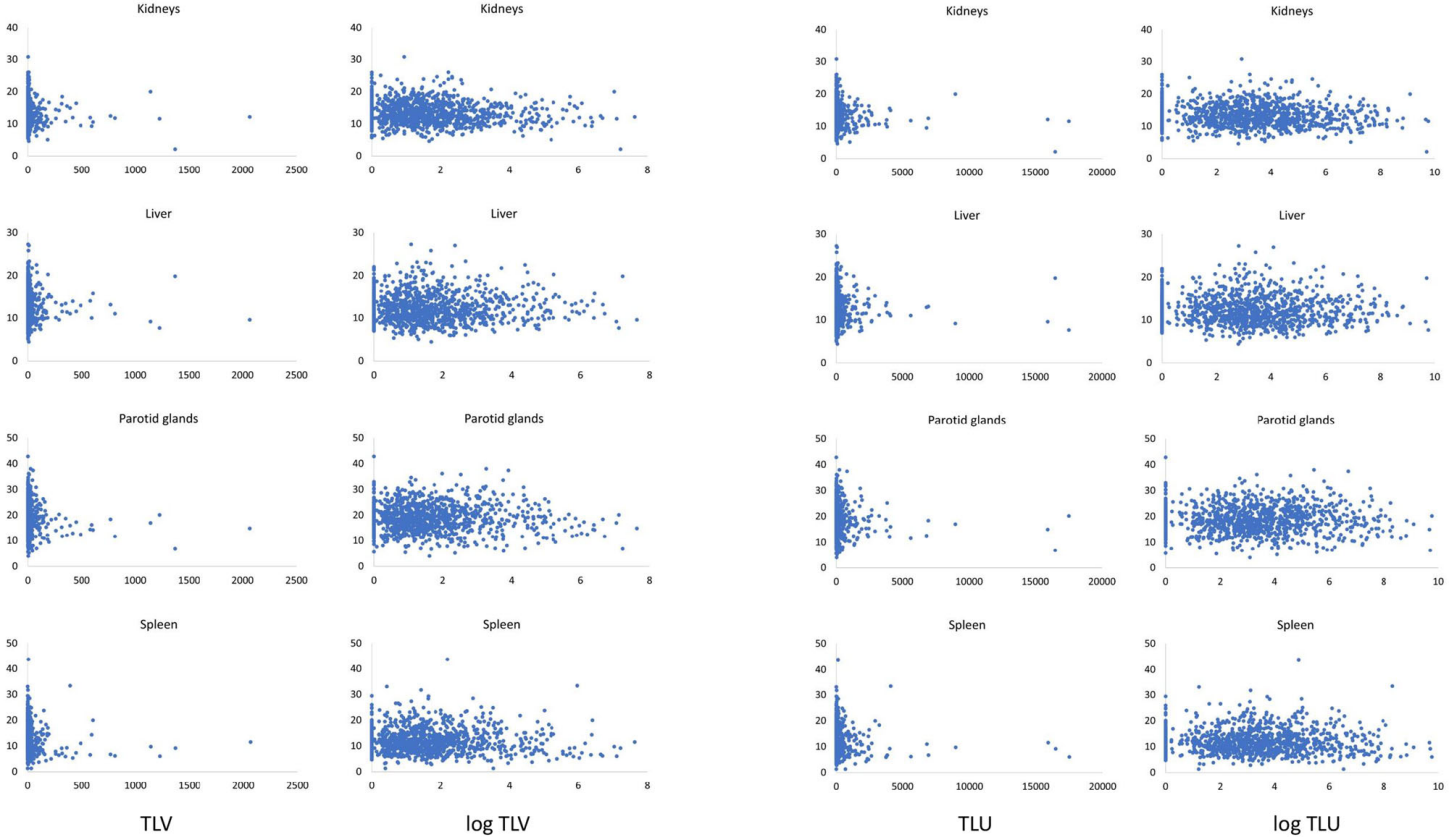
Scatter plots of TLV and TLU (x axis) vs uptake in organs at risk (y axis). The scatter plots show actual values as well as logarithmized values.

**Figure 3:**
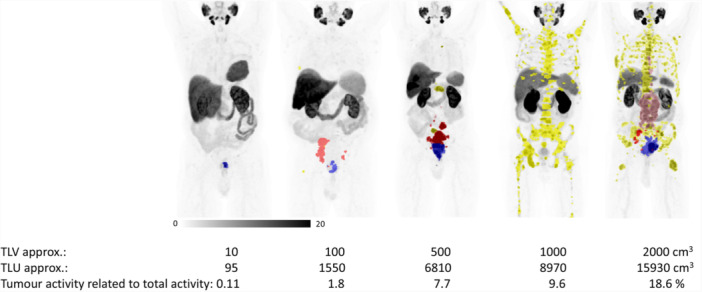
Examples of patients with TLV ranging between 10 and 2000 cm3. The coloured segmentations represent manual segmentations of tumorous lesions (prostate in blue, lymph node metastases in pink/red and bone metastases in yellow).

Scatter plots of the correlation between TLV or TLU and uptake in organs at risk (without and with logarithm) are shown in Figure 2. There was no significant correlation between TLV or TLU and SUV_mean_ in organs at risk (Table 2) for liver, parotid glands or spleen, but a weak negative correlation between TLV/TLU and the SUV_mean_ in the kidneys. Patient examples of different TLV are shown in Figure 3.

**Table 2:** Correlations between tumour burden and uptake in organs at risk.

	TLV	TLU
Kidneys	−0.11 (*p* = 0.0005)	−0.09 (*p* = 0.003)
Liver	−0.02 (*p* = 0.62)	−0.02 (*p* = 0.44)
Parotid glands	−0.007 (*p* = 0.81)	−0.0008 (*p* = 0.98)
Spleen	0.005 (*p* = 0.86)	0.002 (*p* = 0.95)

*Note*: Spearman rho correlation coefficient and *p* values for the correlations between TLV or TLU and SUV_mean_ in organs at risk.

### 3.4 Uptake in organs at risk stratified by TLV groups

**Figure 4:**
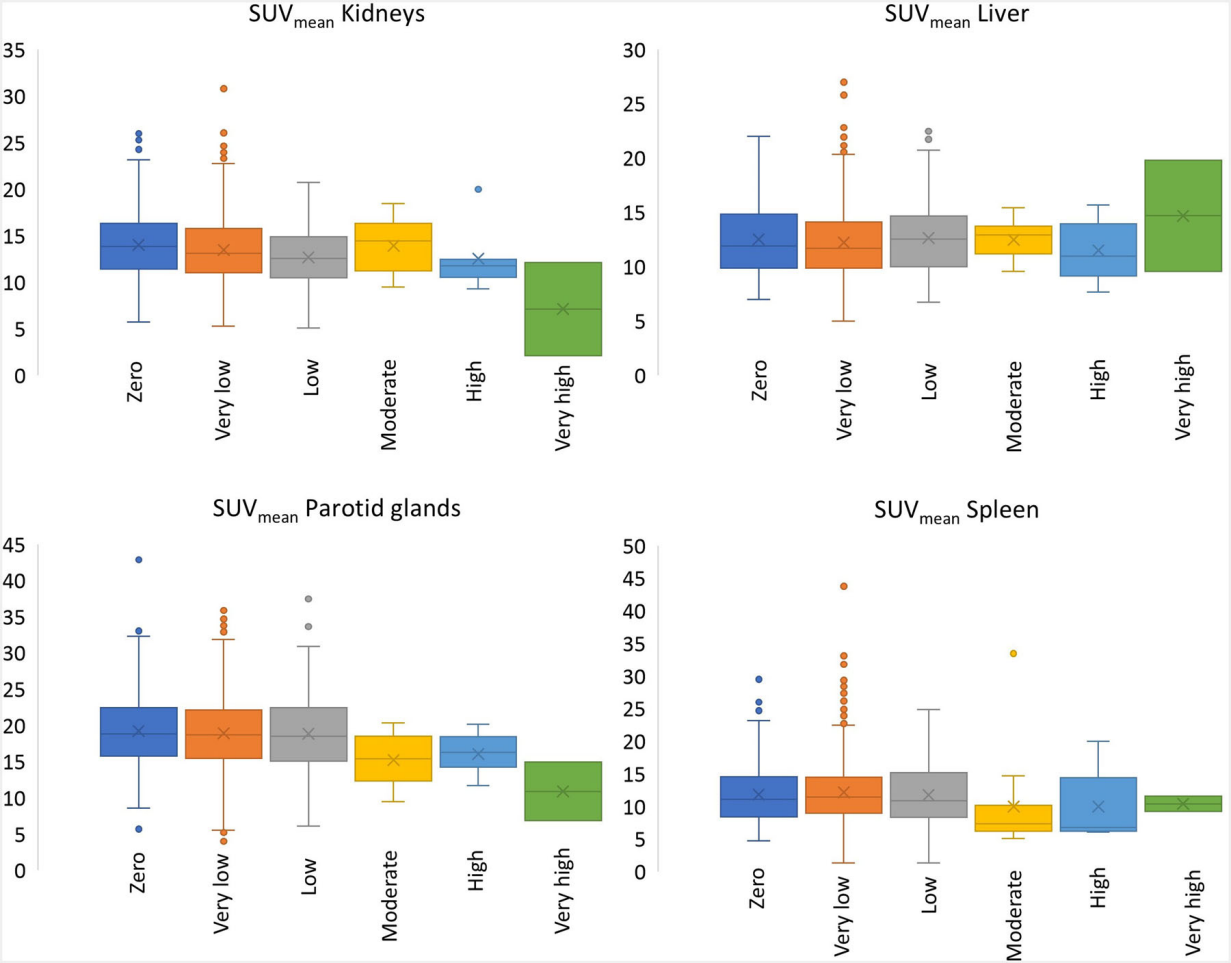
SUVmean of normal reference tissue stratified by TLV.

The number of patients with 0 TLV was *n* = 157, with very low TLV *n* = 801, low TLV *n* = 105, moderate TLV *n* = 13, high TLV *n* = 7 and very high TLV *n* = 2. Figure 4 shows boxplots of the SUV_mean_ of the different organs at risk stratified by TLV. No statistical analyses of difference between the groups were performed due to the very low number of patients in the groups with high and very high TLV.

## 4 DISCUSSION

In this study, the main result was a large uptake variability of [^18^F]PSMA‐1007 in organs at risk. No statistically significant correlations between TLV or TLU and uptake in the organs at risk liver, parotid glands or spleen were found. There was a significant, although weak, negative correlation between TLV/TLU and uptake in the kidneys. The findings were mainly due to the large variability in SUV_mean_ in organs at risk in patients with no or only a small TLV or TLU. However, there was a tendency towards a lower SUV_mean_ in the kidneys and parotid glands in patients with a very high TLV. The percentage of uptake in the tumours relative to the total administered activity was rather low—only three patients had more than 10% of the total administered activity located within the tumours.

Patients being treated with RNT, such as [^177^Lu]Lu‐DOTATATE or [^177^Lu]Lu‐PSMA‐617, are often given a fixed amount of activity. It has been hypothesized that if a tumour sink effect is present, there may be room for higher administered activity for patients with high tumour burden, since the therapeutic activity is limited due to potential toxicity to organs at risk (Yordanova et al., 2017). From the result of our study, it seems that the possible tumour sink effect is rather small and only exists in patients with a very high TLV. This is the largest study on the subject, and to the best of our knowledge, the only study including only [^18^F]PSMA‐1007 images.

Currently, RNT with [^177^Lu]Lu‐PSMA‐617 is mainly provided to patients with metastastic castration‐resistant prostate cancer post taxane and one novel‐generation androgen‐axis targeting drug with a high tumour burden (Kratochwil et al., 2023), i.e. not to the patient population described in this study. However, several studies are ongoing investigating the benefits of using PSMA RNT in earlier disease stages, such as in metastatic hormone‐sensitive prostate cancer or even in localized disease (Jang et al., 2023). Thus, the indication may be relevant also for the present study population in the future.

Controversial results on the relation between uptake in organs at risk and tumour burden in PSMA‐targeted PET have been reported. Gafita et al. (Gafita et al., 2022) conducted a rather large retrospective study (*n* = 406) on patients imaged with [^68^Ga]Ga‐PSMA‐11. Their patients were mainly imaged due to metastatic hormone‐sensitive prostate cancer or metastatic castration‐resistant prostate cancer. They found a moderate negative correlation between TLV and SUV_mean_ of salivary glands (*r* = −0.44), kidneys (*r* = −0.34), and liver (*r* = −0.30) and a weak negative correlation with the spleen (*r* = −0.16). They had a significantly higher TLV than our study (median 302 cm^3^ compared with 2.8 cm^3^ in our study). Correspondingly, they only included 40 patients with TLV = 0 (157 in our study) and only 71 patients with a very low TLV (801 in our study). Thus, there is a possibility that Gafita et al. have underestimated the large variability in SUV_mean_ in normal tissues in patients with no or only very limited tumour burden. Gaertner et al. (Gaertner et al., 2017) found a decline of 36‐43%, 45%, 25%, and 19% of [^68^Ga]Ga‐PSMA‐11 uptake in salivary glands, kidneys, liver, and spleen, respectively, in a patient cohort of 135 individuals with metastatic‐resistant prostate cancer. In their study, the TLV was not measured, but only visually graded. In contrast, Werner et al. (Werner et al., 2020) found a significant correlation between TLV and uptake in the kidneys only. In their study, similar to ours, patients with early‐stage prostate cancer with mainly low tumour burden were included, and the study only included 50 patients.

In this study, no patients being treated with [^177^Lu]Lu‐PSMA RNT were included. Gafita et al. (Gafita et al., 2022) included a small number of patients with metastatic castration‐resistant prostate cancer with a high disease burden on baseline PSMA PET‐CT at the initiation of [^177^Lu]Lu‐PSMA RNT and received a follow‐up scan after two treatment cycles. They found that changes in TLV during [^177^Lu]Lu‐PSMA RNT impact the normal uptake on follow‐up PSMA PET‐CT scans. Hofman et al. (Hofman et al., 2018) have shown that a higher absorbed dose to the tumour was associated with higher rates of PSA response and that TLV on pretherapeutic [^68^Ga]Ga‐PSMA PET was inversely correlated with salivary gland and kidney absorbed radiation dose.

It should be noted that the biodistribution of [^18^F]PSMA‐1007 may not directly reflect the biodistribution of [^177^Lu]Lu‐PSMA‐617. The large variability of the uptake in organs at risk across all TLV groups shows the importance of individual pretherapy dosimetry.

A strength of this study is the large number of patients and the large number of patients with no or with a very low TLV. This made it possible to verify the large variability in SUV_mean_ in organs at risk. It is also a strength that images obtained with [^18^F]PSMA‐1007 were used, as no other such studies have been performed. Additionally, we investigated the influence on TLU, not only TLV, as the previous studies did. Limitations include the low number of patients with high and very high TLV. Also, it is not known if the organs at risk are actually normal. For example, it is known that uptake of [^18^F]PSMA‐1007 in the kidneys is correlated with renal function (Valind et al., 2023). Patients with impaired renal function may exist in the cohort. SUV for large, homogenous tumours/organs can be fairly accurately determined. However, for small tumour volumes, SUV is most likely underestimated due to spatial resolution issues. There was also no standardized way to segment the tumour volumes. Therefore, there is an uncertainty in the determination of the TLU and TLV, especially for small tumours and for cases where the high TLV/TLU were a sum of several small tumours rather than one large tumour.

## 5 CONCLUSIONS

There was a large radiopharmaceutical uptake variability in organs at risk, which demonstrates the need for individual pretherapy dosimetry. No statistically significant correlations between TLV or TLU and SUV_mean_ in the organs at risk liver, parotid glands or spleen were found. There was a significant, although weak, negative correlation between TLV/TLU and uptake in the kidneys. The findings were mainly due to the large variability in SUV_mean_ in organs at risk in patients with no or only a small TLV or TLU. However, there was a tendency towards a lower SUV_mean_ in the kidneys and parotid glands in patients with a very high TLV. The results are not surprising since, in most patients, even in those with a high tumour burden, only a rather small percentage of the total administered activity was located within the tumours.

## AUTHOR CONTRIBUTIONS

All authors contributed to the study conception and design. Material preparation was performed by David Minarik. Development of the CNN was performed by Måns Larsson and Olof Enqvist. Manual segmentation was performed by Elin Trägårdh. Data collection and analysis were performed by Elin Trägårdh, Måns Larsson, David Minarik and Olof Enqvist. The first draft of the manuscript was written by Elin Trägårdh and all authors commented on previous versions of the manuscript. All authors read and approved the final manuscript.

## CONFLICT OF INTEREST STATEMENT

OE and ML are employed by and shareholders of the consultant company Eigenvision AB. The other authors have no financial or nonfinancial interests to disclose. The authors declare no conflicts of interest.

## 1 CONSENT TO PARTICIPATE

Informed consent was obtained from all individual participants included in the study.

## Data Availability

The datasets generated during the current study are available from the corresponding author on reasonable request.
